# The Water Cycle, a Potential Source of the Bacterial Pathogen *Bacillus cereus*

**DOI:** 10.1155/2015/356928

**Published:** 2015-03-30

**Authors:** Julien Brillard, Christian M. S. Dupont, Odile Berge, Claire Dargaignaratz, Stéphanie Oriol-Gagnier, Claude Doussan, Véronique Broussolle, Marina Gillon, Thierry Clavel, Annette Bérard

**Affiliations:** ^1^INRA, UMR 408 Sécurité et Qualité des Produits d'Origine Végétale, 84000 Avignon, France; ^2^Université d'Avignon, UMR 408 Sécurité et Qualité des Produits d'Origine Végétale, 84000 Avignon, France; ^3^INRA-Université Montpellier II, UMR 1333 DGIMI, 34095 Montpellier, France; ^4^CNRS, Université Montpellier II, UMR 5235 DIMNP, 34095 Montpellier, France; ^5^EPIM EA 3647, Université de Versailles St-Quentin-en-Yvelines, 78035 Versailles, France; ^6^INRA, UR 407 Pathologie Végétale, 84140 Montfavet, France; ^7^CNRS, CEA, Université Aix-Marseille, UMR 7265, 13108 Saint-Paul-lez-Durance, France; ^8^INRA, UMR 1114 EMMAH, 84914 Avignon, France; ^9^Université d'Avignon, UMR 1114 EMMAH, 84914 Avignon, France

## Abstract

The behaviour of the sporulating soil-dwelling *Bacillus cereus sensu lato* (*B. cereus sl*) which includes foodborne pathogenic strains has been extensively studied in relation to its various animal hosts. The aim of this environmental study was to investigate the water compartments (rain and soil water, as well as groundwater) closely linked to the primary *B. cereus sl* reservoir, for which available data are limited. *B. cereus sl* was present, primarily as spores, in all of the tested compartments of an agricultural site, including water from rain to groundwater through soil. During rain events, leachates collected after transfer through the soil eventually reached the groundwater and were loaded with *B. cereus sl*. In groundwater samples, newly introduced spores of a *B. cereus* model strain were able to germinate, and vegetative cells arising from this event were detected for up to 50 days. This first *B. cereus sl* investigation in the various types of interrelated environments suggests that the consideration of the aquatic compartment linked to soil and to climatic events should provide a better understanding of *B. cereus sl* ecology and thus be relevant for a more accurate risk assessment of food poisoning caused by *B. cereus sl* pathogenic strains.

## 1. Introduction

The life cycles of many human pathogens, including foodborne pathogens, comprise stages in the environment outside the eukaryotic host, for which data are sometimes limited but are required to monitor adverse effects on human health [1–3]. Ready-to-eat processed foods are usually a combination of multiple ingredients, each bringing its own possibility of contamination by a pathogen into the final food products. The bacterial endospores are among the most resistant forms of living organisms. Their resistance favors their survival to food processing. The multiplication of the vegetative cells formed after spore germination can occur in a wide range of temperatures, pH, or water activities and be the cause of foodborne poisonings [4].

The ubiquitous* B. cereus* group of bacteria, also called* Bacillus cereus sensu lato *(*B. cereus sl*), displays a broad diversity of phylogenetically related strains [5, 6], most of which harbour pathogenic characteristics [7–9]. This group includes closely related species, such as the causative agent of anthrax* B. anthracis*, the entomopathogenic bacterium* B. thuringiensis*, or the human pathogen* B. cereus sensu stricto* (*B. cereus ss*), which is responsible for various human infections (septicaemia, endophthalmitis, periodontitis, etc.) [6, 10]. More importantly,* B. cereus ss* is a common source of foodborne poisoning, displaying emetic or diarrheal syndromes [10, 11] and representing 1 to 33% of cases of foodborne poisonings depending on countries [12]. A study conducted on* B. cereus ss* food poisoning outbreaks in France revealed the emergence of particularly virulent strains and emphasized the danger for public health caused by these bacteria [13], particularly in processed food that is not conserved in appropriate conditions. The sporulating ability of* B. cereus sl* allows these bacteria to survive in various stressful environments, which contributes to their ubiquity [14]. Spores highly resistant to heat and chemical and other stresses can adhere to stainless steel in the food industries despite cleaning-in-place procedures. Accordingly, these properties favour* B. cereus sl* contamination in processed food [14].

Moreover,* B. cereus sl* occurrence has been widely reported in a broad range of environments, such as many types of soils, sediments, dust, and plants (see [8–10, 15] for reviews). Despite increasing numbers of studies of microbiota in atmospheric environments (including water in clouds, fog droplets, etc.) that have regularly identified isolates of* Bacillus* spp. [16–18],* B. cereus sl* has not been specifically described.* Bacillus* occurrence in other natural habitats, such as aquatic environments, is the concern of only a limited number of studies.* B. cereus sl* has been rarely detected in surface fresh water samples [19]. In sediments of an Italian river, two of 83 isolates of sulphite-reducing bacteria were identified as* B. cereus sl* [20]. A few antibiotic-resistant isolates of* B. cereus sl* were also isolated in Chinese aquaculture environments [21]. These scant studies performed in aquatic environments confirm* B. cereus sl* ubiquity. To our knowledge, no specific investigation of* B. cereus sl* in groundwater has yet been performed. Groundwater is a common supply for drinking water, but groundwater is also an important source for irrigation, by flooding (e.g., grassland), by sprinkling (e.g., crops or vegetables), or by drip irrigation (e.g., orchards). If groundwater were contaminated with human pathogens, this practice could disseminate them to cultivated plants.

Despite its distribution in a broad range of natural habitats, little is known concerning how* B. cereus sl* bacteria behave in the environment. Most studies have focused on* B. thuringiensis* because this species is widely spread in the environment as a biological agent for pest control [15, 22] or on* B. anthracis* to elucidate how this species can lead to zoonosis [23]. However, ecological studies of* B. anthracis* have primarily focused on the conditions that lead to transmission of anthrax rather than on the fate of the cells in such environments [24, 25]. For other* B. cereus sl* bacteria, including the human pathogen* B. cereus ss*, many questions remain unanswered: it remains unclear whether* B. cereus ss* grows in bulk soil (whatever its physicochemical properties and nutrient availability) or whether* B. cereus ss *survives in the soil only as dormant spores until particularly favourable microenvironments (niches) are encountered to allow its growth [26]. Growth of* B. cereus sl* in such niches has previously been described, for example, in rhizosphere [27], insect cadavers [15], nematodes [28], arthropod gut microbiota [29], and earthworm guts [30, 31]. In a given environment, there may be a continuous flow of* B. cereus sl* strains with the arrival of new strains originating from elsewhere, whereas others disappear by death or by leaching. Soil is considered a reservoir for* B. cereus sl*; however, soil is not a static medium. Soil is permanently affected by water movement, which redistributes solutes, nutrients, and the geochemical background [32]. Little is known concerning the role of soil water flows during rain events in modifying the pool of* B. cereus sl* strains in soil or concerning the putative transfer of these bacteria to deeper environments and in their fate in groundwater.

A better understanding of* B. cereus sl* ecology should be useful for uncovering the still largely unknown determinants enabling the dispersal and fate of such populations in environmental conditions and might be relevant for a more accurate risk assessment of foodborne poisoning caused by* B. cereus sl* pathogenic strains. The aim of this study was to determine the occurrence and the fate of* B. cereus sl* from rain to groundwater through soil and to investigate a possible bacterial transfer from soil to water compartments. For this purpose, we sampled and isolated* B. cereus sl* in different soil and water compartments of the atmosphere-soil-groundwater continuum in an agroecosystem and performed* B. cereus ss* inoculation survival experiments of soil and water sampled in the same site.

## 2. Materials and Methods

### 2.1. Experimental Area

The experimental area was in south-eastern France at INRA-Avignon (altitude ASL 30 m, lon. 04°53′E, and lat. 43°55′N) 2 km north of the Durance River. The climate is Mediterranean, with a primary wet period in autumn (high intensity storm events) and with severe drought and high temperatures in summer. The various crops grown in this agricultural area require much irrigation water. The soil is composed of clayey silt (2.5 m maximum thickness) overlying an alluvial aquifer consisting of gravels and of sands (7 m depth) on marl bedrock. The water table depth ranges from 4 to 5.5 m [33]. The experimental area (conventional field of 0.6 ha, cultivated in 2010 with sunflower, in 2011 with durum wheat, and in 2012 with corn) is instrumented with 14 piezometers and with one lysimeter. Sampling was performed between April 2011 and April 2012.

### 2.2. Soil

The bulk soil used in this study was collected from the experimental field, as previously described [34], during the intercropping season, several months after the last harvest (June 23, 2011). The soil is a fine textured calcareous silty clay loam (Table 1). Soil samples were used in two approaches: occurrence studies and bacterial inoculation tests.

For* B. cereus sl* occurrence studies, the soil samples were collected in October 2011 from the 0–2, 2–10, 10–20, and 20–30 cm layers of the field and in April 2012 from the 0–2, 2–10, 10–20, 20–30, 30–60, and 60–90 cm layers of the field (Table 2). Surface soil samples (0–2 cm depth) were collected with a cleaned stainless steel trowel; the other soil samples (<2 cm depth) were vertically collected by coring with a cleaned stainless steel auger. Soil samples were placed in clean plastic bags and stored at 4°C before* B. cereus sl* measurements (performed within 7 days). Substrate-induced respiration (SIR) with glucose was performed using the MicroResp technique [34], which is a rapid microtiter plate method to measure carbon dioxide evolved from carbon substrate amendments to determine the physiological profiles of soil microbial communities by using whole soil. The SIR microbial measurement is considered a good indicator of the total physiologically active bacteria in soil, which is a proxy of the active microbial biomass [35]. The data of* B. cereus sl* occurrence in soil were analysed using Student's *t*-test to determine *P* values for differences in expected values versus actual values.

For* B. cereus ss* inoculation experiments, the soil samples were collected in March 2012 from the 0–10 cm layer of the field, air-dried, and sieved into a size fraction between 2 and 3 mm. After sampling, drying, and sieving, the soil was slowly moistened and maintained at 21% of the gravimetric water content (a value equivalent to the water content usually observed in this soil at this time of the year and also optimal for microbial activities) and stored for seven days at room temperature before inoculation experiments [36].

### 2.3. Rainwater

Rainwater was collected on the site as follows: when rain was about to fall or when the rainfall event had recently begun, an autoclaved plastic bag (50 L) was placed in a circular 21 L bucket with a diameter of 0.30 m (corresponding to a surface area of 0.071 m^2^). The bucket was held on a stick 2 m above the ground to avoid any risk of contamination by rain splashing on the soil. The bucket was in the middle of the experimental field, 25 m from the closest hedge to limit the risk of contamination by neighbouring vegetation. The rainwater was collected 3–18 h later and immediately filtered on a 0.2 *μ*m pore size membrane. Then, the particles, including putative bacteria located on the filter, were suspended in 2 mL of filtered rainwater, giving a 100–200-fold concentration of the rain sample, depending on the volume of rainwater collected. The rain samples used for* B. cereus sl* occurrence studies were collected in October 2011, November 2011, March 2012, and April 2012. During all of these rainfall events, the dominant winds were south-easterly.

### 2.4. Soil Percolated Water

A lysimeter placed in a monolith of undisturbed soil (1.8 m × 2.6 m surface area and 2.2 m depth) permanently exposed to natural climatic conditions was at the north-west end of the field-site. Cultivated identical to the nearby field, the lysimeter is dedicated to the measurement and calculation of soil water fluxes at the plot scale, with a continuous monitoring of hydrographs of water drainage events [37]. The bottom of the lysimeter was equipped with drains and with a fluxmeter for the automatic recording of the amount of percolated water (one automatic measurement for every 20 g of water drained). The lysimeter was constructed in 2003 and had since been under crop rotation identical to the field next to the experimental site. A device was set up to collect, in sterile containers, leachate samples drawn from the drains of the lysimeter for periods of several hours, depending on the percolated water fluxes. Percolated water collected during five rain events from April 2011 to April 2012 was concentrated as described above for rainwater (Section 2.3). The recorded hydrographs of drained water enabled the calculation of the mean water flow rate and the period of drainage (except for April 2011, for which hydrodynamic data were not available). The mean* B. cereus sl* flow during the sampling period was calculated.

### 2.5. Groundwater

Groundwater was collected as follows: a piezometer, which tapped the upper part of the saturated zone (depth 6 m) and was in the south-east of the area, was hand-sampled using an immerged pump after the complete renewal of the water in the piezometer. Groundwater, which was collected in a sterile plastic bag, was concentrated as described above for rainwater (Section 2.3). The borehole was sampled four times in autumn 2011 and in spring 2012. All water samples presented the same features: the pH ranged between 7.1 and 7.2; the temperature was equal to 16 ± 0.1°C; and electrical conductivity, characterising the mineralisation of groundwater, ranged between 654 and 658 *μ*S/cm. Previous analyses of groundwater (~60 samples) in several boreholes near the study area from 2009 to 2011 highlighted the predominance of Ca^2+^ and HCO_3_^−^ in solution. The concentration of organic carbon in groundwater, which may be present as a particulate or dissolved, ranged between 0.4 and 9.6 mg/L, with a mean concentration equal to 1.3 mg/L.

### 2.6. Strains and Growth Conditions

#### 2.6.1. Isolation and Enumeration of* B. cereus sl *from Environmental Samples

For the isolation, identification, and counts of* B. cereus sl *CFU in these environmental samples with the presence of background microbiota, we used a selective medium recommended by food authorities. The Mossel medium (also called MYP for mannitol-egg yolk-phenol red-polymyxin-agar), which was purchased from Biokar Diagnostics (Beauvais, France), allows the growth of* B. cereus sl* bacteria [38] and their identification as lecithinase-positive and mannitol-negative colonies. Because of the existence of rare lecithinase-negative or mannitol-positive* B. cereus sl* strains that were discarded in these conditions, our bacterial counts might be slightly underestimated [38]. We used 10% TSA medium (Trypticase soy agar, Difco) to count culturable aerobic heterotrophs after incubation for 48–72 h at 20°C.

Aliquots of 0.25 g of soil were supplemented with 1 mL of sterile saline water and vortexed vigorously for 30 s. The suspension was serially diluted 10-fold in sterile saline water, and 100 *μ*L of each dilution was plated in triplicate on Mossel medium and incubated for 24 h at 30°C. The variability between triplicates did not exceed 0.26 log CFU, indicating that the bacterial counts were reproducible. For determining the* B. cereus* spore density, the samples were pasteurised for 20 min at 70°C to eliminate vegetative cells and then incubated on selective medium. These thermoresistant bacteria were counted as spore forms because only spores of* B. cereus sl* survive at this temperature.

#### 2.6.2. Inoculation of Environmental Samples with a* B. cereus ss* Model Strain

For the inoculation of environmental samples, the R2SK model strain for human pathogenic* B. cereus ss* strains was used. This strain is a spectinomycin- (Spc-) resistant strain derived from the ATCC 14579 strain [39]. The minimal temperature of growth for this mesophilic strain is 10°C [40]. Growth was routinely performed at 30°C on Luria Bertani (LB) medium (Difco), unless otherwise stated. LB-Spc (275 *μ*g/mL) was used for counting this strain after the inoculation of natural samples.

*B. cereus *R2SK spores, which were prepared on the FNA sporulation medium [41] for 7 days at 25°C, were used for the inoculation of natural samples. These spores were washed three times in cold sterile saline (0.9% NaCl) water, pasteurised for 20 min at 70°C to remove any remaining vegetative cells, and stored at 4°C until use.

*B. cereus *R2SK vegetative cells used for the inoculation of natural samples were grown in Gordon's medium containing soil extracts [42]; however, only the first soil infusion step was performed. The medium was inoculated with 1% of an overnight culture of R2SK strain, and growth was performed at 30°C with shaking (200 rpm) until OD_600 nm_ = 3.0. Then, the cells were washed twice in sterile saline water at 20°C before soil or water inoculation.

Soil aliquots (0.25 g) introduced into sterile 2 mL screw-cap microtubes were inoculated with 20 *μ*L of* B. cereus *R2SK vegetative cells to reach approximately 4 log CFU/g (a level below that of the natural* B. cereus sl* population in soil) and incubated at 25°C in the dark. Counting was performed over time as described above for the enumeration of the natural* B. cereus sl *population (Section 2.6.1); however, serial dilutions were plated in triplicate on LB-Spc. The first count was performed immediately after inoculation, and a second count was performed one hour later to confirm that no rapid changes in bacterial counts had occurred, which could be caused, for instance, by the dissociation of long chains of vegetative cells or of putative spore aggregates, as previously described [43]. To determine the spore content, the initial suspension was rapidly (<1 min) transferred at 70°C to a water-bath for 20 min as described above (Section 2.6.1).

Groundwater aliquots (100 mL) were inoculated with 1 mL of* B. cereus* R2SK spores or of vegetative cells, which were prepared as described above, and incubated at 12°C in the dark without shaking.

When required, populations of* B. cereus* R2SK that were below the detection threshold (10 CFU/mL) were arbitrarily set at 9 CFU/mL, as previously described [40].

### 2.7. Diversity of* B. cereus sl* Natural Isolates

Some isolates (*n* = 3) originating from rainwater that were defined as* B. cereus sl* bacteria after growth on Mossel medium were characterised, as previously described [44], using API50CH (BioMérieux), which is a kit that allows the determination of bacterial respiration on 50 different carbohydrates [45] to confirm that the bacteria did belong to* B. cereus sl.* In addition, sequencing of the* rrs* gene was performed after a PCR amplification on 5 randomly selected strains (*n* = 3 from rain and *n* = 2 from groundwater), as previously described [6]. Briefly,* rrs* genes were amplified by PCR using the primers 5-AGA GTT TGA TC(A,C) TGG CTC AG 3-(forward primer) and 5-GG(A,C) TAC CTT GTT ACG A(T,C)T TC 3-(reverse primer), using the following temperature profile: 30 cycles of 94°C for 1 min, 58°C for 1 min, and 72°C for 2 min, followed by a final extension at 72°C for 5 min. The amplification products were purified and DNA-sequenced by Eurogentec. A BLASTN search was performed against nr/nt database to confirm that best hits (with 100% query coverage and 97–100% identity) were obtained with* B. cereus sl* sequences. These methods are powerful tools for identifying* B. cereus sl* but are not able to discriminate isolates at the species level.

The diversity of the* B. cereus sl* natural isolates was assessed using a fingerprint method (M13 PCR), as previously described [46, 47], on 80 randomly selected isolates collected from soil samples originating from various depths (*n* = 40) and from percolated water (*n* = 40). The images of the PCR fragments were analysed using the Biogen 99.04 software (Vilber Lourmat, Marne-la-Vallée, France) for band detection, binary matrix generation, similarity coefficient calculation, and dendrogram construction. Images were also sight-checked to clear unspecific bands detected by the software. Similar visual patterns were obtained between duplicates. A control strain (ATCC14579) was always used, which showed >85% identity in 15 independent analyses. Thus, genuine distinct isolates were distinguished from putative clones when the percentage similarities were <85%.

## 3. Results

### 3.1. Isolation of* B. cereus sl* Strains from Rainwater

The occurrence of* B. cereus sl* ubiquitous bacteria has never been studied in rain and was investigated in this study.

**Figure 1:**
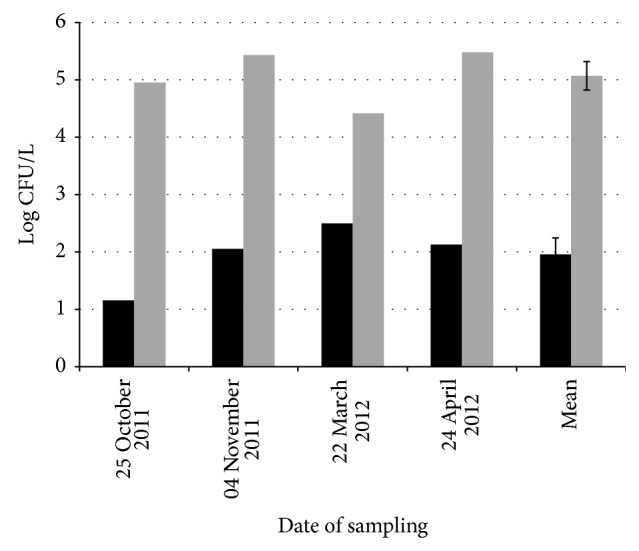
Bacteria isolated from rainwater. CFU counts were performed in four independent rain samples. The calculated mean values ± SEM from these four samples are also indicated. Grey bars: total culturable aerobic heterotrophs; black bars:* B. cereus sl* bacteria.

Our results (Figure 1) indicate that the level of total culturable bacteria ranged from 4.41 to 5.48 log CFU/L in the four rainwater samples (mean ± SEM 5.07 ± 0.25 log CFU/L). The investigated samples always contained culturable* B. cereus sl* at densities ranging from 1.16 to 2.50 log CFU/L (mean ± SEM 1.96 ± 0.28* B. cereus sl* log CFU/L), 2 to 4 log lower than the total culturable bacteria. Both the phenotypic characterisation (using an API50CH kit) and sequencing of the* rrs* gene, which were performed on a few isolates, confirmed that the isolates were genuine* B. cereus sl* isolates.

### 3.2. Occurrence of* B. cereus sl* in an Agricultural Soil

*B. cereus sl* bacteria are commonly isolated from soils. The level of the natural culturable* B. cereus sl* population was investigated in the soil used during our experiments.

As expected,* B. cereus sl* bacteria were present in all of the soil samples, with a population ranging between 4.37 and 5.23 log* B. cereus sl*/g of soil (Table 3). The mean densities ± SEM per gram of soil of total* B. cereus sl* and thermoresistant* B. cereus sl* CFU (i.e., spores) were not significantly different (4.92 ± 0.09 versus 5.06 ± 0.04 log CFU/g, *P* > 0.05), indicating that* B. cereus sl* was almost exclusively present as spores in the soil tested.

The different seasons did not seem to modify the occurrence of the natural* B. cereus sl* population of this soil; the mean density ± SEM per gram of soil displayed no significant difference between samples collected in October and in April (5.09 ± 0.04 versus 4.92 ± 0.08 log CFU/g, *P* > 0.05).

In addition, the amount of* B. cereus sl* isolated did not seem to be influenced by the depth of soil in our sampling conditions. For instance, the mean* B. cereus sl* densities ± SEM per gram of soil were not significantly different between the superficial horizon (0–20 cm: 5.01 ± 0.06 log CFU/g) and the deeper depths (20–90 cm: 4.95 ± 0.09 log CFU/g, *P* > 0.05).

The decrease of values obtained using SIR measurements with the depth of soil indicates a higher active microbial biomass content in the upper layers of soil investigated (Table 2).

Altogether, these data suggest that the* B. cereus sl* population consisted nearly exclusively of dormant spores in the tested soil and was not affected by the season or by the soil depth (down to 90 cm) (Table 3).

### 3.3. *B. cereus sl* Isolated from Soil Leaching through the Lysimeter

*B. cereus sl* was isolated from all of the samples of water that percolated through the soil in the lysimeter; however, the density of its population and its estimated flux (mean during sampling period) varied (Table 4). We investigated thermoresistant* B. cereus sl* CFU (i.e., spores) in the three leaching water samples from 2012, and their densities were similar to those densities of the total* B. cereus sl* CFU (mean values: 3.76 and 3.69 log CFU/L, resp.), suggesting that* B. cereus sl* was almost exclusively present as spores in water soil leachates.

During the November 2011 rainfall event, six distinct fractions of the leachates were collected, three at the beginning and three at the end of the leaching event. The mean fluxes of* B. cereus sl* isolated during the beginning of the leaching event were significantly higher than during the end of the same leaching event (4.97 ± 0.39 versus 3.15 ± 0.27* B. cereus sl* log CFU/L, mean ± SEM, *P* < 0.05, Student's *t*-test) (Table 4). At the time scale of a single rainfall event and at the megascopic scale of the lysimeter, the bacteria density or flux increases with the water flux of the drained water.

### 3.4. *B. cereus sl* Isolated from Groundwater

Samples of groundwater were collected and investigated for the presence of* B. cereus sl* (Table 5). These samples were collected during a rainfall event (6 April 2012) or several weeks later (13 October 2011).

Three of the four samples indicated the presence of culturable* B. cereus sl*. In these three samples, the number of CFUs was equal to the detection threshold value, suggesting that only a low density of* B. cereus sl* was present in the studied groundwater samples.

### 3.5. Diversity of Environmental Isolates

**Figure 2:**
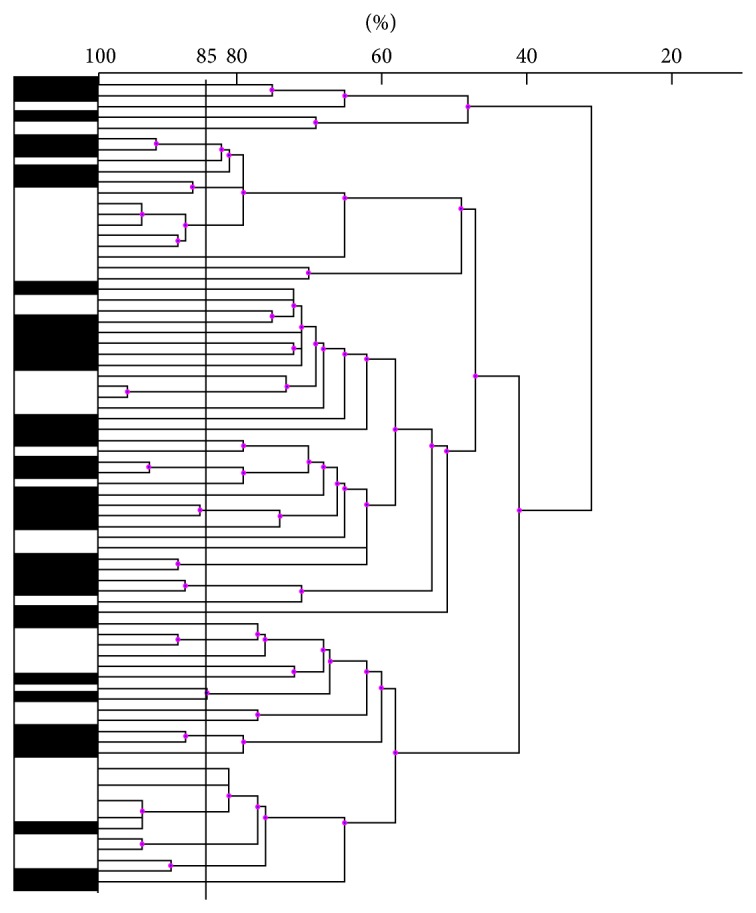
Dendrogram illustrating the diversity of* B. cereus sl* natural isolates. M13-PCR was performed on 40 isolates from soil (black boxes) and on 40 isolates from percolated water (white boxes). Genuine distinct isolates were distinguished from putative clones when the percentage similarity (indicated above) was <85% (black vertical bar).

To obtain an overview of the diversity of* B. cereus sl* natural isolates from soil samples, a fingerprint method was performed on 40 isolates (originating from various depths). To determine whether some groups of strains could be preferentially leached out from soil, the method was also applied on 40 isolates collected from percolated water (Figure 2).

High diversity was observed among the isolates of* B. cereus sl*, with many of them displaying different fingerprint profiles. Such diversity was observed for both soil and percolated water isolates. Among 80 isolates tested, at least 56 were considered distinct (displaying a similarity <85%). The dendrogram did not reveal any specific group of isolates from soil or from percolated water.

### 3.6. Behaviour of* B. cereus ss *in Aliquots of Soil and of Water

We monitored how a newly introduced model* B. cereus ss* population evolved over time in aliquots of soil and of groundwater that contained their natural microbiota. The water samples originating from rain and from percolated water were not investigated in this study because they were considered transient environments for* B. cereus sl*.

#### 3.6.1. Behaviour of* B. cereus ss *in Aliquots of Soil

**Figure 3:**
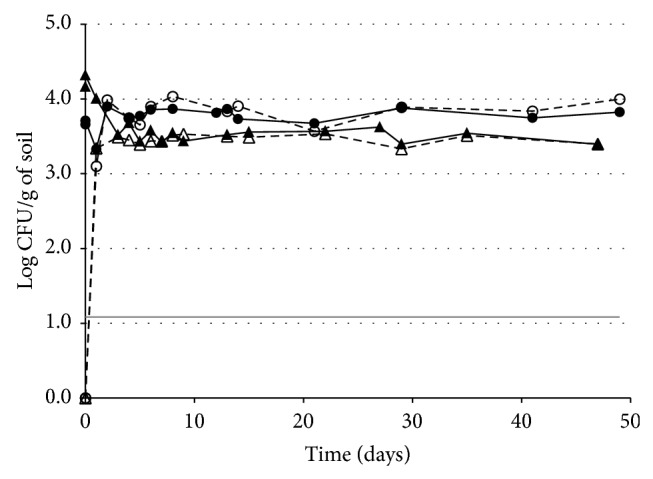
Fate of* B. cereus ss* vegetative cells introduced into the soil. Counts of CFUs and thermoresistant CFUs (i.e., spores) of* B. cereus ss* were performed over time. Two independent experiments (triangles, replicate 1; circles, replicate 2) are presented. Full symbols and full lines:* B. cereus ss* total CFU; open symbols and dashed lines:* B. cereus ss* thermoresistant CFU; grey horizontal line: detection threshold (1.08 log CFU/g). The data for the spores at time 0 were below the detection threshold but were arbitrarily set at 1 CFU/g (i.e., 0 log CFU/g) because growth conditions for the preparation of the inoculum were not appropriate to allow sporulation.

The natural* B. cereus sl* population of the soil used for inoculation experiments was 4.89 log CFU/g (mean value of duplicate experiments, range 4.82–4.94). Soil samples were inoculated by* B. cereus ss* vegetative cells of the Spc-resistant R2SK strain at a density between 3.66 and 4.16 log CFU/g, slightly below that of the natural* B. cereus sl* population level in this soil. A noninoculated control soil sample displayed no detectable Spc-resistant CFUs. Counts performed over time showed a rapid decrease of approximately 0.5–1 log in the newly introduced population during the first three days following inoculation (Figure 3). This decrease was concomitant with the appearance of thermoresistant* B. cereus* R2SK CFU (i.e., spores).

After this period, the remaining newly introduced* B. cereus* R2SK population was represented only by spores. This population reached a steady state, with no further changes in the level of* B. cereus* R2SK population observed up to 50 days of incubation at 25°C. Soil samples inoculated with vegetative cells (at 7.8 log CFU/g) of* B. cereus* R2SK still displayed high levels of thermoresistant* B. cereus* R2SK CFU (6.9 log CFU/g) after a 10-month period of storage in the dark at room temperature (not shown). This result indicates that the spores were extremely stable in this environment.

In addition, no major change in the level of the* B. cereus* R2SK population was observed over time when the soil was inoculated with spores instead of with vegetative cells (not shown).

#### 3.6.2. Behaviour of* B. cereus ss *in Aliquots of Water

*(1) In Groundwater Inoculated with Vegetative Cells.* Aliquots of groundwater were inoculated with* B. cereus ss* strain R2SK vegetative cells at 8.20 or 8.21 log CFU/L, a density largely above that usually encountered in this groundwater. Counts of the newly introduced population were performed over time. In the tested samples, the natural* B. cereus sl* population was below the detection threshold (3.48 log CFU/L).

**Figure 4:**
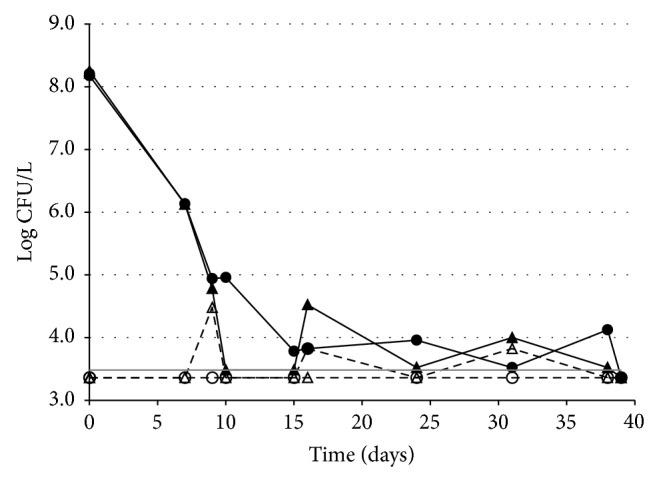
Fate of* B. cereus ss* vegetative cells introduced in groundwater. Counts of CFUs and thermoresistant CFUs (i.e., spores) of* B. cereus ss* were performed over time. Two independent experiments (triangles, replicate 1; circles, replicate 2) are presented. Full symbols and full lines:* B. cereus ss* total CFU; open symbols and dashed lines:* B. cereus ss* thermoresistant CFU; grey horizontal line: detection threshold (3.48 log CFU/L). The data below the detection threshold were arbitrarily set at 3.36 log CFU/L. (This value corresponds to the maximum CFU value possible below the detection limit.)

After inoculation, the newly introduced vegetative cells of* B. cereus* R2SK showed a high constant death rate during the first 10–15 days (Figure 4). After this period, the total* B. cereus* R2SK population reached a low density ranging between the detection threshold and 4 log CFU/L. The level of this surviving* B. cereus* R2SK population did not significantly change over time until 38 days of incubation, after which the density of the viable* B. cereus* R2SK cells decreased below the detection threshold. Thermoresistant* B. cereus* R2SK CFUs were occasionally detected at levels slightly above the detection threshold, indicating that* B. cereus* R2SK cells could sporulate under these conditions or that sporulation may have occurred before inoculation at a level below the detection threshold.

**Figure 5:**
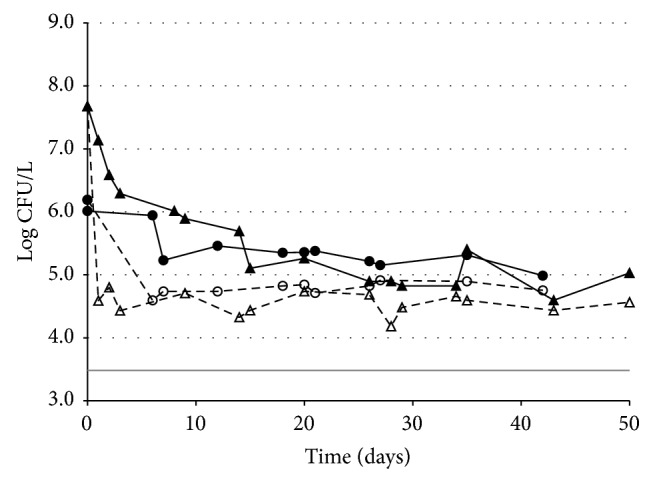
Fate of* B. cereus ss* spores introduced in groundwater. Counts of CFUs and thermoresistant CFUs (i.e., spores) were performed over time. Two independent experiments (triangles, replicate 1; circles, replicate 2) are presented. Full symbols and full lines:* B. cereus ss* total CFU; open symbols and dashed lines:* B. cereus ss* thermoresistant CFU; grey horizontal line: detection threshold (3.48 log CFU/L).

*(2) In Groundwater Inoculated with Spores*. Groundwater was inoculated with spores of* B. cereus* strain R2SK at 7.67 or 6.19 log CFU/L (a density largely above that usually encountered in this groundwater in natural conditions but a density of only 1 to 3 log higher than the highest density encountered in lysimeter leachates), and counts were performed over time (Figure 5). A rapid decrease in the number of thermoresistant CFUs was observed during the first few days, reaching a steady density of approximately 4.5–5 log CFU/L after less than five days. The number of remaining viable spores seemed extremely stable over time (for up to 40–50 days after inoculation). These results suggest that a proportion of any of the spore population reaching the groundwater environment could persist there for a long time.

The total culturable* B. cereus* R2SK population (i.e., spores plus vegetative cells) also displayed a rapid decrease during the first days after inoculation. However, the decrease was slower than that observed for the counted spores alone. This result indicates that rapid (but incomplete) germination occurred in the groundwater tested. For up to 20 days of incubation, the total* B. cereus* R2SK population reached a density of approximately 5–5.5 log CFU/L, which was at least 1 log CFU/L higher than the density of spores, indicating that a significant proportion of the* B. cereus* R2SK was viable as vegetative cells in this environment. The presence of vegetative cells was confirmed by microscopic observations (data not shown). Then, this total* B. cereus ss* population decreased slightly over time, up to 40–50 days, but was still at a higher density than spores. Under these conditions, the vegetative cells seemed well adapted to this groundwater environment, because these cells were able to survive for at least 50 days.

## 4. Discussion

*Bacillus cereus sl* are soil dwelling bacteria, which include foodborne pathogenic strains, that are able to adapt to a wide range of environments. However, little is known concerning the occurrence, fluxes, or fate of* B. cereus sl* bacteria in various aquatic environments (rain, leachates, or groundwater) that are connected to the main reservoir of these bacteria (i.e., soil).

### 4.1. *B. cereus sl* Is Present in Rainwater

In recent years, an entire microbial ecosystem has been identified in the atmospheric environment, including rain and fog water [16–18, 48], particularly through the development of large-scale molecular methods (metagenomic approaches). To our knowledge,* B. cereus sl* has not been described as a regular member of this microbial ecosystem, whereas the spore-formers* Bacillus* spp. were observed in the atmosphere [17] and during dust events [49, 50]. We detected* B. cereus sl* in each of the four rain samples collected 2 m above ground level. Thus,* B. cereus sl* bacteria appeared to belong to the normal microbiota of rain. Delort et al. reviewed several studies of microorganisms found in atmospheric water (including rainwater) [51]. Among three major studies, the levels of total bacteria ranged from 6 to 9 log/L (by microscopic cell count); the authors also stated that less than 1% of the collected bacteria could be cultivated on nonselective media [51]. These results are in agreement with the CFU count in the rainwater collected during our study. Similarly, we cannot rule out the possibility that an additional viable but nonculturable* B. cereus sl* population is present in our samples. However, whether* B. cereus sl* occurs in rain drops as free spores or attached to dust particles remains to be investigated. It would be interesting to determine whether* B. cereus sl* found in raindrops originates from soil dust that has been aerosolized and is potentially associated with long-distance dispersal, as suggested by Kellogg and Griffin [49], or directly originates as spores that may have reached the stratosphere with ascending airflows, as already suggested for other bacteria [52].

### 4.2. Regular Occurrence and Behaviour of* B. cereus sl* in an Agricultural Soil

#### 4.2.1. *B. cereus sl* Is Present in Soil as Spores

As hypothesised, we identified* B. cereus sl* strains in the investigated agricultural soil, at a level similar to those levels previously described in other soils [22, 53]. This level seemed constant during two different seasons and at various soil depths. Usually, total bacterial numbers in soil are considered to decrease with depth, because of the decrease in carbon availability [54]. This possibility was confirmed by our microrespirometric data showing a higher active microbial biomass content in the upper layers of the soil investigated. In the soil studied in this research, the natural* B. cereus sl* soil population consisted almost exclusively of dormant spores. Spores do not depend on available organic matter to survive, and this finding may explain why the numbers of* B. cereus sl* did not decrease with depth similar to total bacteria. The data for* B. cereus sl* bacteria in relation to soil depth are scant. A previous study showed that a* B. thuringiensis* population introduced on the surface of a soil by spraying six years before counting significantly decreased at a depth below 10 cm [30]; however, the density of natural* B. cereus sl* isolates at various depths was not investigated.

The observed shift of a* B. cereus ss* population from vegetative cells to spores when introduced into a nonfavourable environment, such as the raw soil used in this study, is in agreement with previous studies [30, 43, 55]. Spores that arose from the newly introduced* B. cereus ss* model population seemed to settle efficiently in the tested soil, with their level remaining highly stable over time, up to ten months. Experimental data concerning the long-term survival of* B. cereus sl* spores are scant.* B. anthracis* was shown to stay viable in a slurry of soil for six months under laboratory-controlled conditions [31], and* B. thuringiensis* was able to survive for at least thirteen years in the field [56].

#### 4.2.2. Lack of Observed* B. cereus ss* Growth in Soil

Previous studies suggest that* B. cereus sl* spores become active when an excess of easily decomposable organic matter is bioavailable or when the soil presents a high moisture level [57]. In our study, the counts failed to reveal any major increase in the newly introduced* B. cereus ss* model population in this bare agricultural soil; however, this soil was not highly moistened, was not enriched with nutrients, and contained its own microbiota. We also investigated the fate of the introduced* B. cereus ss* population in similar soil samples “cleared” of their own microbiota by treatment with chloroform fumigation, and no change in the level of the newly introduced* B. cereus ss* population was observed (Brillard and Bérard, unpublished data). This finding suggests that a putative competition with the natural microbiota of the tested soil is not responsible for the rapid sporulation of the introduced* B. cereus ss* population.

#### 4.2.3. Determinants Allowing* B. cereus sl* Multiplication

Because the* B. cereus sl* population in the studied soil was constant over time (at 6-month intervals) and because rainwater that permits reinoculation of soil contained a lower density of* B. cereus sl* population than percolated water, causing its elimination, other sources of bacteria or conditions allowing* B. cereus sl* multiplication (i.e., “hot spots” of organic matter) must sometimes occur. The term “incubator area” describes the hypothesis of Van Ness, whereby certain soil conditions may favour* B. cereus sl* vegetative growth in “hot spots” of microbial activities, such as the rhizosphere [26]. For instance, it was previously shown that the numbers of* Bacillus* CFUs counted in different rhizosphere samples were 1 log higher than in the control soil without plants [58]. Roots and mycorrhizal exudates represent a major source of dissolved sugars, amino acids, and other organic acids in soil [59], providing highly heterogeneous microenvironments [60] and driving the structure and dynamics of microbial communities [61, 62]. At least 6 free amino acids are able to trigger* B. cereus sl* germination [63] and may be present in such enriched environments. Moreover, alternatives to sporulation were described in studies showing that* B. anthracis* forms biofilms and persists as a vegetative form in a model rhizosphere system [27, 31]. The soil used for* B. cereus ss* inoculation experiments was collected in March 2012, several months after the last cultivated plant (durum wheat harvested in June 2011). This soil was quite poor in organic matter (organic carbon content 13.2 g kg^−1^) and roots compared with some grassy meadow (36.1 g kg^−1^) or forest soils, for instance [34], providing an environment less favourable for the multiplication of the* B. cereus ss* population. Additionally, the favourable conditions for* B. cereus ss* growth most likely occur at microspatial and microtemporal scales, which explain the stability and homogeneity of our larger scale* in situ* observations.

More ecological studies are required to clarify the environmental determinants allowing* B. cereus sl* growth in soil with different strains (using isolates originating from this soil, for instance), different soils, and different incubation conditions (e.g., higher humidity, addition of different sources of carbon, etc.), or in association with a rhizosphere or with soil organisms to test the effect of physicochemical conditions and to provide new sources of organic matter for the growth, germination, survival, and sporulation of these populations.

### 4.3. *B. cereus sl* Can Be Leached Out of Soil and May Reach Groundwater

#### 4.3.1. *B. cereus sl* Is Present in Leachates

The* B. cereus sl* density in percolated water was up to 3 log higher than that encountered in rainwater, suggesting that* B. cereus sl* CFUs were leached out of soil by water seeping through the ground. Given the size of bacteria (a few microns) and the fact that bacteria are most often adsorbed onto larger soil particles, most of the transport process of bacteria in soil would occur in the largest pore sizes of the soil [64, 65]. The largest pore sizes are also where the preferential flow, bypassing the soil matrix, occurs under natural soil conditions (e.g., [66]). Such preferential flows during high rainfall intensity events have been shown previously in this lysimeter and piezometers of the experimental site [33, 37]. The spore exosporium is believed to play a key part in its attachment to soil substrates and, therefore, is assumed to restrict spore dispersal [24]. In this study, the spores may still be attached to or even encompassed into small soil particles during leaching events. Additional knowledge concerning the* B. cereus sl* attachment and detachment processes [64, 67] should improve our understanding concerning the transport of these bacteria from soil to groundwater.

#### 4.3.2. *B. cereus sl* May Reach Groundwater

In this experimental field context, the aquifer is located only a few meters below the percolated water collection lysimeter equipment. Thus, our data led us to reasonably hypothesize that* B. cereus sl* can be transferred from soil to groundwater via leachates. We observed* B. cereus sl* in some of our groundwater samples but at an extremely low density. A sandy gravel layer of 2-3 m can be found between soil and groundwater. Pore size exclusion phenomena rather than hydrophobic interactions between spores and soil organic content seem to play an important role in the filtration process in soils [65, 68]. However, an earlier study investigating infiltration processes in the same experimental site suggested that preferential flows observed during high rain events had a quantitative effect on this alluvial aquifer recharge in only isolated cases but could induce local short-term contamination of the groundwater [33].

The groundwater sample that did not display any* B. cereus sl* was sampled in spring 2012 after an extremely dry winter (37 mm between December 2011 and April 2012). The* B. cereus sl* density in water samples and flows observed from the lysimeter leachates were lower during the April 2012 rain event than during 2011 rain events. Garel et al. [33] suggested that the soil capacity to transfer water downwards depends on the rainfall features and on the prior soil moisture conditions because the preferential water flows through the soil depend on the initial water content (and on the saturation of soil macropores) [69]; under wet conditions, the rainfall event of November 2011 (163 mm) may have had an effect on groundwater, whereas in a drier historic context the lower rainfall event of April 2012 (50 mm) may have had less influence on the groundwater. Therefore,* B. cereus sl* occurrence in groundwater could be linked to climatic conditions and seasons (after heavy rain in a wet context, e.g.).

#### 4.3.3. Fate of* B. cereus ss* in Groundwater

Because* B. cereus sl* may reach groundwater after being leached out of soil, the survival, germination, or even growth could occur in this aquatic environment.

Introduced in groundwater samples as vegetative cells, the model strain of* B. cereus ss* primarily died rapidly; however, a small part remained viable over time. These results suggest that only a minority of the vegetative cells previously grown on rich laboratory media were adapted to survive in a low-nutrient environment such as this groundwater sample. In contrast, a significant part of the* B. cereus ss* population introduced as spores in these groundwater samples survived. Interestingly, germination occurred, and vegetative cells arising from these spore germination events seemed well adapted to this low-nutrient environment, being able to survive for at least 50 days. Nutritional cues for spore germination may be present as traces in groundwater and could explain the observed* B. cereus sl* germination in such environment. For instance, several free amino acids are known to trigger* B. cereus sl* germination [63]. A different hypothesis would be that the germination is triggered by biological signals emitted by bacterivorous protists inhabiting groundwater. Recent data indicate that, in response to factors excreted by amoebae,* B. cereus sl* can escape ingestion but can also germinate and grow [70, 71].

Because of the rapid decrease in* B. cereus ss* counts observed during our experiments and because of a high detection threshold, groundwater samples were inoculated at high densities, (i.e., spores were inoculated at a density of 1 to 3 log higher than the highest density encountered in lysimeter leachates). Thus, our results only provide an overview of the possible fate of* B. cereus ss* in this environment but should require more investigation to decipher what occurs under more natural conditions. Taken together, these data suggest that a proportion of any spore that reaches the groundwater environment may persist there for a long time as both spores and vegetative cells.

Thus, the occurrence of* B. cereus sl* in groundwater, even at low levels, may not be only temporary. Therefore, risks of the dissemination of* B. cereus sl* via groundwater should be considered in future ecological studies of these bacteria.

### 4.4. Environmental Isolates Are Diverse

The high diversity of* B. cereus sl* isolates originating from soil, as observed by a fingerprint method, is consistent with previous results showing much diversity among the* B. cereus sl* population in temperate soils [72–75]. The agricultural field area investigated in this study had never been treated by* B. thuringiensis* (as a pest control agent); however, such utilisation may have occurred in the neighbouring agricultural area, and we cannot exclude the possibility of contamination by* B. thuringiensis *strains [76]. However, the high diversity of isolates originating from soil suggests that a major occurrence of the* B. thuringiensis* pest control clonal strain in our soil samples can be ruled out.

No particular group of isolates was identified in our lysimeter percolated samples, suggesting that no particular strains could be leached out of the soil. However, additional investigations should be performed on* B. cereus sl* diversity (in terms of functional and pathogenic traits) in environmental samples linked to transport among the unsaturated zones of the soil for a better risk assessment of ground water contamination.

## 5. Conclusions

To our knowledge, this study provides a first description of the fate and transport of* B. cereus,* which is a foodborne pathogen, in rain, soil, soil leachate, and groundwater in the context of a field study. Despite using culturable methods that are expected to underestimate the densities of bacterial populations, the presence of* B. cereus sl* was detected in various environmental samples, including rain and groundwater compartments, which have rarely been described. In the investigated soil, the* B. cereus sl* population seemed constant over time; however, after high rain periods,* B. cereus sl* was leached out of soil suggesting that* B. cereus sl* may be transferred to groundwater, where it could persist for a long period.

This descriptive study also opens new fields for future research. This study revealed or confirmed that more ecological studies are required (i) to determine the origin of* B. cereus sl *strains isolated in rain samples, (ii) to clarify the determinants allowing* B. cereus sl* growth in the main reservoir (i.e., soils), (iii) to understand the attachment and detachment processes to/from the soil matrix linked to rain events, (iv) to specify the role of the various ground layers in probable filtration of bacteria before they could reach groundwater, and, finally, (v) to determine the adaptive potential of human pathogenic strains to new environments compared with nonpathogenic ones, given the high diversity of strains observed in environmental samples. The possible fate of* B. cereus sl* in groundwater should however require more investigation to decipher what occurs under more natural conditions (i.e., with lower cell densities).

**Figure 6:**
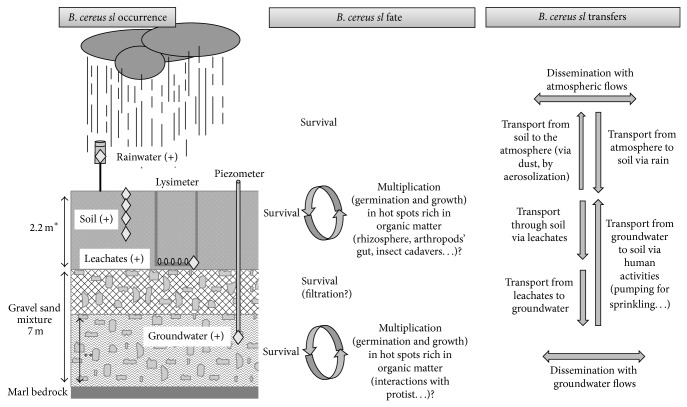
Schematic representation of the lysimeter plot and a proposed model of* B. cereus sl* occurrence, fate, and transfers in the agricultural site. The occurrence of* B. cereus sl* was investigated during this study (white diamonds) and observed primarily as spores in the indicated compartments (“+” symbol).  ^*^Height of the lysimeter. The soil layer can be slightly deeper or thinner elsewhere on the agricultural plot.  ^**^Water table depth ranges from 4 to 5.5 m, depending on the season.

Depending on the type of food, it was proposed that the contamination by* B. cereus sl* through soil is rarely the main or the only one [4]. Although the question of* B. cereus sl* transfer to foods via diverse routes and at diverse steps of food processing requires more investigation, this study brings new insights about* B. cereus sl* occurrence and behavior in the environment, especially into the water cycle, which may represent yet unstudied routes for food contamination. A proposed model of a possible part of the* B. cereus sl* life cycle is represented in Figure 6. Soil is a reservoir of dormant spores of* B. cereus sl.* When particularly favorable conditions are encountered (presumably at specific spatial and temporal scales), growth may occur so that the pool of* B. cereus sl* is maintained in the soil. Therefore, soil may provide a source for the occasional contamination of rainwater (possibly via air dust) and of groundwater (in which* B. cereus sl* may survive) via leachates and, consequently, could contribute to* B. cereus sl* propagation in distant environments. Thus, rain and groundwater should be considered when studying the* B. cereus sl* life cycle in the environment, particularly in the context of agroecosystems submitted to climatic changes (i.e., heavy rains). Considering these environmental sources of* B. cereus sl *may be helpful for the more accurate assessment of the risk of food poisoning, particularly in ready-to-eat food, by pathogenic strains of* B. cereus sl.*

## Figures and Tables

**Table 1:** Selected physicochemical characteristics of the soil sampled (0–10 cm depth) for *B. cereus sl* inoculation experiments.

Soil physicochemical characteristic	Value
Clay (g kg^−1^)^*^	323
Silt (g kg^−1^)^*^	259
Sand (g kg^−1^)^*^	45
Water hold capacity (% moisture content)	31.4
pH (water)	8.51
CaCO_3_ (g kg^−1^)	347
Cation-exchange capacity (cmol kg^−1^)	11.4
Total organic carbon (g kg^−1^)^*^	13.2
Total organic matter (g kg^−1^)^*^	22.8
Total nitrogen (g kg^−1^)^*^	1.54
*C* : *N* ratio	8.59

^*^After decarbonatation.

**Table 2:** Selected physicochemical and microbiological characteristics of the soil layers sampled in this study.

Depth of soil sampling (cm)	October 2011		April 2012			
	SIR-microbial biomass (*μ*g C g^−1^ soil)	Humidity (% g DW soil)^*^	SIR-microbial biomass (*μ*g C g^−1^ soil)	Humidity (% g DW soil)	NO_3_^−^ (mg kg^−1^ soil)	pH-water
0–2	90	5.5	202	3.7	83.9	8.29
2–10	67	15.3	150	16.7	88.9	8.44
10–20	56	16.2	163	17.4	89.7	8.52
20–30	36	16.7	165	18.6	73.4	8.38
30–60	ND	ND	22	20.1	61.9	8.46
60–90	ND	ND	10	17.8	51.5	8.61

^*^DW: dry weight.

**Table 3:** Occurrence of *B. cereus sl* in an agricultural soil^*^.

Depth of soil sampling	October 2011		April 2012	
	Total *B. cereus sl* log⁡CFU/g of soil	Thermoresistant *B. cereus sl* log⁡CFU/g of soil	Total *B. cereus sl* log⁡CFU/g of soil	Thermoresistant *B. cereus sl* log⁡CFU/g of soil
0–2 cm	5.00	5.22	4.43	4.95
2–10 cm	5.05	5.23	4.91	4.95
10–20 cm	4.98	5.07	5.23	5.15
20–30 cm	5.16	5.02	5.02	5.06
30–60 cm	ND^**^	ND	5.04	5.08
60–90 cm	ND	ND	4.37	4.88

^*^Values from triplicate measurements of the samples (see Section 2).

^**^ND: not done.

**Table 4:** Lysimeter leaching events: *B. cereus sl* density in water percolated from soil. Water flows during sampling periods. *B. cereus sl* flows estimated during sampling periods.

Date of rain event	Rainwater (mm)	Dates of water sampling	*B. cereus sl* (log⁡CFU/L)	Mean water flow during the sampling period (L/h)	Mean *B. cereus sl* flow during the sampling period (log⁡CFU/h)
25-26 April 2011	33.5	25 April 2011	5.48	ND^***^	ND
01–04 June 2011	61.5	4 June 2011	5.69	0.698	5.53
01–07 November 2011	183	3 November 2011^*^	5.18	0.187	4.46
		3-4 November 2011^*^	4.98	0.551	4.72
		4 November 2011^*^	5.46	1.940	5.75
		9-10 November 2011^**^	3.95	0.266	3.38
		10–14 November 2011^**^	4.26	0.163	3.47
		14 November 2011^**^	3.48	0.134	2.61
03-04 April 2012	40	5 April 2012	3.62	0.049	2.31
		5-6 April 2012	3.66	0.027	2.09
10 April 2012	10	10–12 April 2012	3.83	0.006	1.61

^*^Measurements performed at the beginning of the November 2011 rain-leaching event.

^**^Measurements performed at the end of the November 2011 rain-leaching event.

^***^ND: not done.

**Table 5:** *B. cereus sl* CFU isolated from groundwater.

Date of water sampling	*B. cereus sl* log⁡CFU/L^*^	Previous rainfall event
13 October 2011	0.17 (0.17)	06 August 2011 (54.5 mm)
		03 September 2011 (42 mm)
14 November 2011	0.96 (0.96)	01–07 November 2011 (163 mm)
06 April 2012	Below limit of detection (<0.96)	03–10 April 2012 (50 mm)^**^
18 April 2012	0.43 (0.43)	03–10 April 2012 (50 mm)

^*^The detection threshold is indicated in brackets and depends on the volume of water initially sampled.

^**^First significant rainfall event after several months (previous rainfall event in November 2011).

## Abbreviations

*B. cereus sl*: * Bacillus cereus sensu lato*
*B. cereus ss*: * Bacillus cereus sensu stricto*
DW: Dry weight
SEM: Standard error of the mean
SIR: Substrate-induced respiration
Spc: Spectinomycin
LB: Luria Bertani
CFU: Colony forming unit
TSA: Trypticase soy agar
MYP: Mannitol-egg yolk-phenol red-polymyxin-agar.
